# Supplementary material for: State-dependent energy conversion produces degenerate dissipation in active actomyosin networks
Source: bioRxiv. 2026 Jun 28:2026.04.22.720181. Preprint. [Version 3] doi: 10.64898/2026.04.22.720181 (PMC13320965; doi:10.64898/2026.04.22.720181)
Supplement: Supplement 1 [file media-1.pdf]

Supplementary Materials for : **State-dependent energy conversion produces degenerate dissipation in active actomyosin networks**

Zachary Gao Sun[1,2,3,4], Juanjuan Zheng[4], A. Pasha Tabatabai[2,5], Joost J. Vlassak[4], and Michael Murrell[1,2,3,5,*]

[1] Department of Physics, Yale University, 217 Prospect Street, New Haven, Connecticut 06511, USA

[2] Systems Biology Institute, Yale University, 850 West Campus Drive, West Haven, Connecticut, 06516, USA

[3] Integrated Graduate Program in Physical and Engineering Biology, Yale University, New Haven, Connecticut 06520, USA

[4] John A. Paulson School of Engineering and Applied Sciences, Harvard University, Cambridge, MA, USA

[5] Department of Biomedical Engineering, Yale University, 55 Prospect Street, New Haven, Connecticut 06511, USA

[*]Corresponding author. Email address: michael.murrell@yale.edu .

Table of Contents

Experimental Details 3

Equations & Calculations 5

Catch bond & Slip bond binding rate: 5

Kinetic equations 5

Actin polymerization 5

Myosin ATP hydrolysis 5

ATP regeneration system 6

Mechanical power calculation using PIV velocity field derived from visco-elastic dissipation 8

Derivation from $v(r,t)$ to strain-rate tensor $Dij(r,t)$ to PSD $Sxxf$. 9

Supplementary figures 11

SFig 1. Statistical test for monotonicity. 11

SFig 2. Actomyosin heat dissipation control experiments. 12

SFig 3. Effective unbinding rate plot. 13

SFig 4. Crossover frequency. 14

SFig 5. Mechanical dissipation and efficiency plot. 15

References 16

Supplementary movie captions 17

Supplementary Movie 1. 2D Crosslinked actin network contraction under myosin active stress. 17

Supplementary Movie 2. 3D fascin-crosslinked actomyosin network local deformation. 17

Experimental Details

Microscopy and rheology experiment buffer conditions

Rheology experiment:

Table 1.

Actin overnight depolymerization:

	Skeletal muscle actin acetone powder	ATP	Tris HCl	DTT	NaN_3	$CaCl_2$	pH
Final conc.	10 mg/mL (238µM)	0.2 mM	2 mM	0.2 mM	0.005%	0.2 mM	7.0

Table 2.

Actin polymerization on rheometer:

	Skeletal muscle actin	ATP	Tris HCl	$MgCl_2$	KCl	$CaCl_2$	crosslinker
Final conc.	1.5625 mg/mL (37.5 µM)	6.25 mM	2 mM	2 mM	100 mM	0.2 mM	$R_c * C_{actin}$

Microscopy experiment:

Table 3.

Actin overnight depolymerization:

	Skeletal muscle actin acetone powder + rhodamine labeled actin (total 15% labeled)	ATP	Tris HCl	DTT	NaN_3	$CaCl_2$	pH
Final conc.	10 mg/mL (238µM)	0.2 mM	2 mM	0.2 mM	0.005%	0.2 mM	7.0

Table 4.

Actin polymerization in chamber:

	Skeletal muscle actin (15% rhodamine labeled)	ATP	Tris HCl	$MgCl_2$	KCl	50x Glucose Oxidase & Catalase*	50x Glucose*	$CaCl_2$	crosslinker

Final conc.	1.5625 mg/mL (37.5 μM)	0.5 mM	2 mM	2 mM	100 mM	6 μL	6 μL	0.2 mM	$R_c * C_{actin}$

*Note:

1. 50x Glucose Oxidase & Catalase is a mixture of Glucose Oxidase (CALBIOCHEM) of 5mg/mL with Catalase (CALBIOCHEM) of 0.9mg/mL[1,2]. (0.1 mg/mL and 0.018 mg/mL in experimental chamber respectively).

2. 50x Glucose contains 5 mg/mL of D-glucose mixed with 0.5% 2-mercaptoethanol (βME)[1,2]. (0.1mg/mL in experimental chamber).

Equations & Calculations

Catch bond & Slip bond binding rate:

The binding kinetics of the two types of bonds can be described as follow:

For slip bond:

$$k_{off}(f) = \frac{1}{\tau_{c0}} e^{\frac{f \, \Delta x_c}{k_B \, T}} \tag{1}$$

, and for catch bond:

$$k_{off}(f) = \frac{1}{\tau_{c0}} e^{-\frac{f \, \Delta x_c}{k_B \, T}} \tag{2}$$

.

Kinetic equations

Actin polymerization

Actin polymerization follows the following kinetic equation[3]:

$$A_m \cdot ATP + end \rightleftharpoons A_F \cdot ATP(end) \rightleftharpoons A_F \cdot ADP \cdot P_i + \Delta Q \tag{3}$$

, in which A_m is G-actin, A_F F-actin, end filament end, and P_i phosphate released from ATP hydrolysis.

Myosin ATP hydrolysis:

$$A \cdot M + ATP \rightharpoonup A + M \cdot ATP \tag{4}$$

$$M \cdot ATP \rightleftharpoons M \cdot ADP \cdot P_i + A \rightleftharpoons A \cdot M \cdot ADP \cdot P_i \rightharpoonup A \cdot M \cdot ADP + P_i + \Delta Q \tag{5}$$

, in which A is actin, M myosin. Eqn. (4) shows myosin detachment from actin through ATP binding, and eqn. (5) shows the myosin power stroke and heat dissipation ΔQ during P_i release, where chemical free energy is converted into mechanical work or stress and dissipated as heat.

ATP regeneration system (PH-LDH +PEP).

$$ATP + H_2O \rightharpoonup ADP + P_i \quad (6)$$

$$ADP + PEP \rightharpoonup ATP + pyruvate \quad (7)$$

, which leaves the net reaction to be:

$$PEP + H_2O \rightharpoonup pyruvate + P_i \quad (8)$$

Chamber volume V = 80 nL.

ATP regeneration via pyruvate kinase (PK-LDH).

Net reaction per ATP turnover: PEP + H_2O → pyruvate + P_i.

Use |ΔG_net| ≈ 60 kJ·mol^{-1}.

Heat dissipation rate during the process:

$$\dot{Q} = c_{head} \cdot r \cdot V \cdot 10^{-6} \cdot \Delta G$$

, with units:

c_head [µM] · r [s^{-1}] · V [L] · ΔG [J·mol^{-1}] × (10^{-6} mol·L^{-1} per µM).

Converting volume to nL and power to nW yields:

$$\dot{Q}[nW] = c_{head} \cdot r \cdot V_{nL} \cdot \Delta G_{kJ} \ / \ 1000$$

For V_{nL} = 80 and ΔG_{kJ} = 60:

$$coefficient = \frac{(80 \times 60)}{1000} = 4.8$$

Per turnover:

Per ATP:

$q = \frac{\Delta G}{N_A}$ ≈ 60,000 J·mol^{-1} / 6.022×10^{23} ≈ 1.03×10^{-19} J.

Table A — Dissipation and inferred $c_{head} \cdot r$ (ΔG = 60 kJ·mol^{-1})

myosin:actin (R_{myo})	Dissipation P (nW)	c_head × r (µM·s^{-1})
0.0002	0.53	0.110

0.002	0.47	0.098
0.006	1.19	0.248
0.02	1.50	0.312
0.05	1.08	0.225
0.1	0.42	0.087

At constant myosin concentration ($R_{myo} = 0.02$):

1) Actin only

Condition	$\dot{Q}$ (nW)	c_head × r ($\mu M \cdot s^{-1}$)
Actin only	1.2469	0.2598

2) Fascin-crosslinked network

Fascin:Actin	$\dot{Q}$ (nW)	c_head × r ($\mu M \cdot s^{-1}$)
1e-05	1.1823	0.2463
0.0001	1.0904	0.2272
0.001	0.7666	0.1597
0.01	0.7089	0.1477
0.1	0.6551	0.1365

3) α-Actinin–crosslinked network

α-Actinin:Actin	$\dot{Q}$ (nW)	c_head × r ($\mu M \cdot s^{-1}$)
1e-05	1.3147	0.2739
0.0001	1.2281	0.2559
0.001	1.3377	0.2787
0.01	1.2243	0.2551
0.05	1.0158	0.2116

Mechanical power calculation using PIV velocity field derived from visco-elastic dissipation

1) Linear viscoelastic stress–strain relation

In shear, the stress is related to strain history by Boltzmann superposition:

$$\sigma(t) = \int_{-\infty}^{t} G(t-\tau)\ \dot{\gamma}(\tau)\, d$$

In frequency space:

$$\sigma(\omega) = G^{*}(\omega)\, \gamma(\omega), \quad G^{*} = G' + iG''$$

For sinusoidal strain $\gamma(t)=\gamma_0 \sin(\omega t)$, one finds:

$$\langle p_{mech} \rangle = \frac{1}{2}\, \omega\, \gamma_0^2\, G''(\omega)$$

Since

$$\Gamma(\omega) = \int_{-\infty}^{\infty} \gamma(t) e^{-i\omega t} dt$$

Parseval/Wiener-Khintchine theorem gives:

$$\frac{1}{2\pi T} |\Gamma(\omega)|^2 = S_{\gamma}^{(2)}(\omega)$$

Here $S_{\gamma}^{(2)}(\omega)$ is the two-sided PSD, and T the averaging window.

Since $f = \frac{\omega}{2\pi}$,

$$S_{\gamma}(f) = 2 S_{\gamma}^{(2)}(2\pi f)$$

, and

$$< \gamma^2 > = \int_{0}^{\infty} S_{\gamma}(f)\, df$$

.

Using $S_{\gamma}(f)$ for one-sided PSD ($f \geq 0$, $\omega = 2\pi f$):

$$\langle p_{mech} \rangle = \int_{0}^{\infty} [\, 2\pi f\, G''(2\pi f)\,]\ S_{\gamma}(f)\, df$$

Since $S_{\dot{\gamma}}(f) = (2\pi\, f)^2 S_{\gamma}(f)$:

$$\langle p_{mech} \rangle = \int_{0}^{\infty} \frac{G''(2\pi f)}{2\pi f}\, S_{\dot{\gamma}}(f)\, df$$

Defining $\eta'(\omega) = \frac{G''(\omega)}{\omega}$, this is:

$$\langle p_{mech} \rangle = \int_0^{\infty} \eta'(2\pi f)\; S_{\dot{\gamma}}(f)\, df$$

For a 2D/3D velocity field, using the strain-rate tensor $D_{ij}(\vec{r}, t)$ and its PSDs:

$$\langle p_{mech} \rangle \approx 2\beta \int_0^{f_{max}} \eta'(2\pi f)\; \left[S_{xx}(f) + S_{yy}(f) + 2S_{xy}(f)\right] df$$

Derivation from $\boldsymbol{v(\vec{r}, t)}$ to strain-rate tensor $\boldsymbol{D_{ij}(\vec{r}, t)}$ to PSD $\boldsymbol{S_{xx}(f)}$.

Strain-rate tensor from $v(\vec{r}, t)$:

$$D_{ij}(\vec{r}, t) \;=\; \frac{1}{2}\,(\partial_i\, v_j(\vec{r}, t) \;+\; \partial_j\, v_i(\vec{r}, t))$$

Key components:

$$D_{xx} \;=\; \partial_x\, v_x,\;\; D_{yy} \;=\; \partial_y\, v_y,\;\; D_{xy} \;=\; \frac{1}{2}\,(\partial_x\, v_y \;+\; \partial_y\, v_x)$$

Temporal Fourier transforms at a pixel (two-sided, angular frequency ω):

$$V_j(\vec{r}, \omega) \;=\; \int_{-\infty}^{\infty} v_j(\vec{r}, t) e^{-i\omega\, t}\, dt$$

$$D_{ij}(\vec{r}, \omega) \;=\; \int_{-\infty}^{\infty} D_{ij}(\vec{r}, t)\; e^{-i\omega\, t}\, dt$$

Spatial derivatives commute with the temporal FT:

$$D_{ij}(\vec{r}, \omega) \;=\; \frac{1}{2}\,(\partial_i\, V_j(\vec{r}, \omega) \;+\; \partial_j\, V_i(\vec{r}, \omega))$$

For the xx component:

$$D_{xx}(\vec{r}, \omega) \;=\; \partial_x\, V_x(\vec{r}, \omega)$$

Two-sided temporal PSD at a pixel (window T):

$${S_{xx}}^{2}(\vec{r}, \omega) \;=\; \frac{1}{2\pi\, T}\; |D_{xx}(\vec{r}, \omega)|^2$$

Parseval consistency (time average):

$$\left\langle {D_{xx}}^2(\vec{r},t)\right\rangle_t \ = \ \frac{1}{2\pi}\int_{-\infty}^{\infty} {S_{xx}}^2(\vec{r},\omega)\ d\omega$$

One-sided PSD in Hz ($f \geq 0$, $\omega = 2\pi f$), with spatial averaging over Ω:

$$S_{D_{xx}}(\vec{r},f) \ = \ 2\ {S_{D_{xx}}}^2(\vec{r},2\pi f)$$

$$S_{xx}(f) \ = \ \left\langle\ S_{D_{xx}}(\vec{r},f)\right\rangle_{\boldsymbol{x}\in\Omega}$$

Supplementary figures

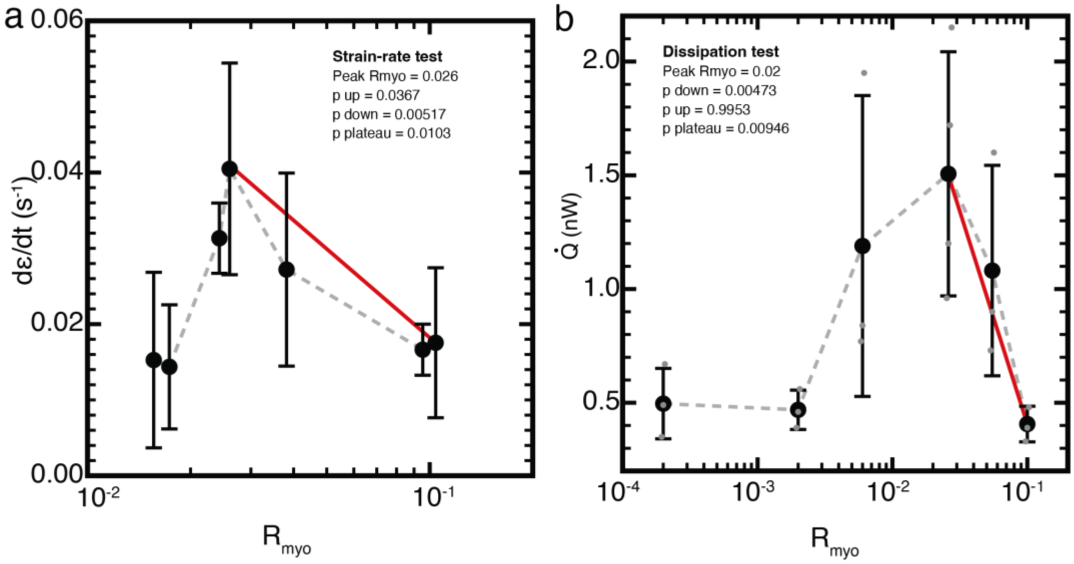

SFig 1. Statistical test for monotonicity. a) Strain rate, dε/dt, plotted as a function of myosin concentration ratio, R_{myo}. Data are shown as mean ± standard deviation. The gray dashed line connects the mean values. The red line shows the fitted post-peak trend after the maximum at R_{myo}= 0.026. The post-peak strain-rate trend significantly decreased with increasing R_{myo} (p_{down} = 0.00517) and rejected a plateau/no-slope relationship ($p_{plateau}$= 0.0103). **b)** Heat dissipation rate, Q̇, plotted as a function of R_myo. Gray points show individual measurements, black points show mean ± standard deviation, and the gray dashed line connects the mean values. The red line shows the fitted (linear model least squares regression) post-peak trend after the maximum at R_{myo}= 0.02. Post-peak dissipation significantly decreased with increasing R_{myo} (p_{down} = 0.00473) and rejected a plateau/no-slope relationship ($p_{plateau}$ = 0.00946).

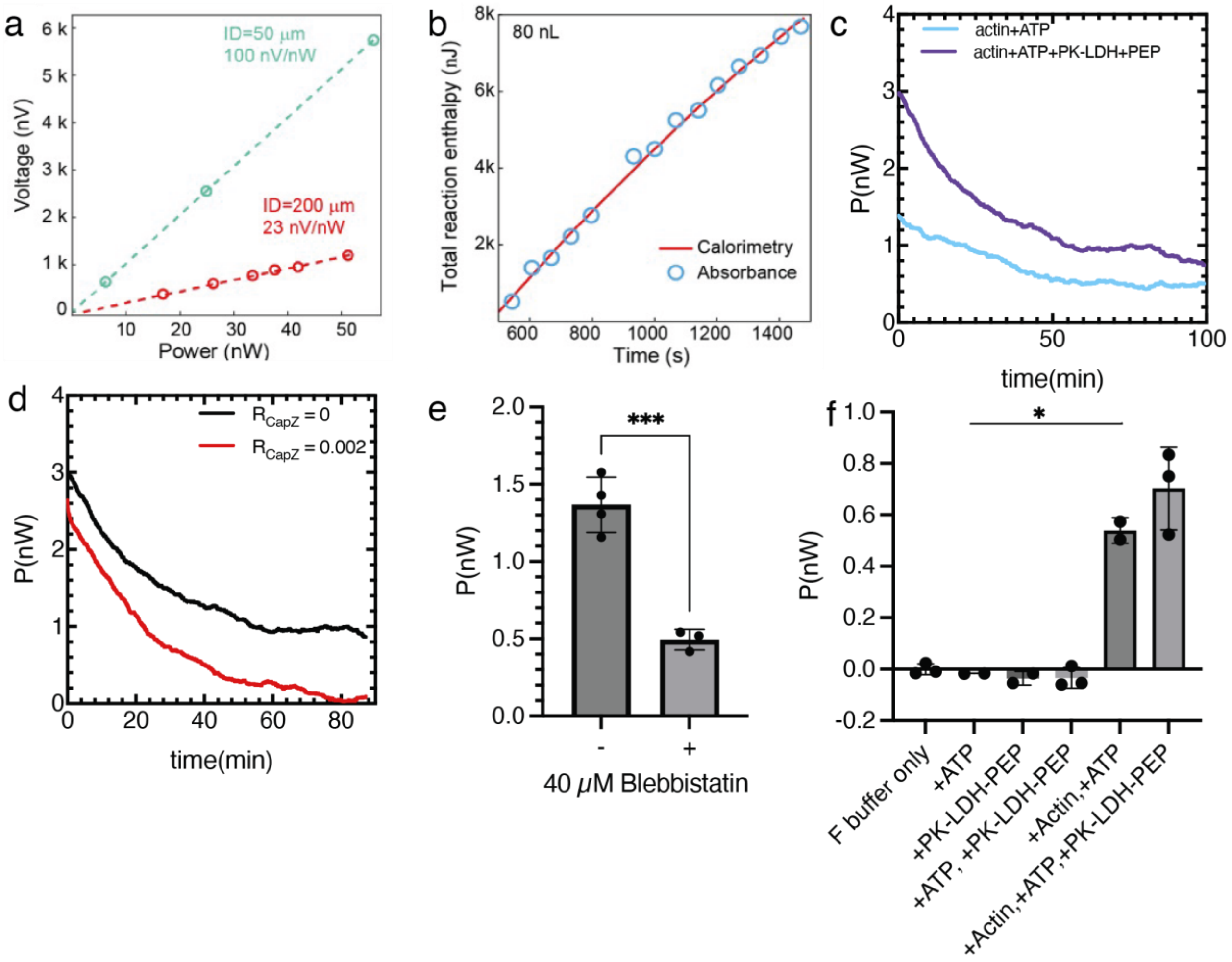

SFig 2. Actomyosin heat dissipation control experiments. a) Electrical calibration: Thermopile voltage response as a function of power applied to the on-chip heating element for sensors assembled with 50 μm-ID and 200 μm-ID capillaries. The 200 μm-ID configuration was used in this work of Zheng et al., *PNAS*, 2026[4]. b) Chemical calibration: Total reaction enthalpy measured calorimetrically compared with values obtained from NADH absorbance measurements for an 80 nL reaction volume[4]. c) Heat dissipation rate P as a function of time for actin polymerization with ATP only (blue) and actin with ATP + ATP regeneration (purple). d) Heat dissipation rate P as a function of time for actin polymerization with (red) and without (black) capping protein CapZ. e) Steady state heat dissipation rate P of actomyosin system without (-) and with (+) blebbistatin. N = 4 and 3 respectively. $p = 0.0008$. f) Steady state heat dissipation rate P of various control cases. N = 3,2,2,3,2,3 respectively. . $p = 0.0390$.

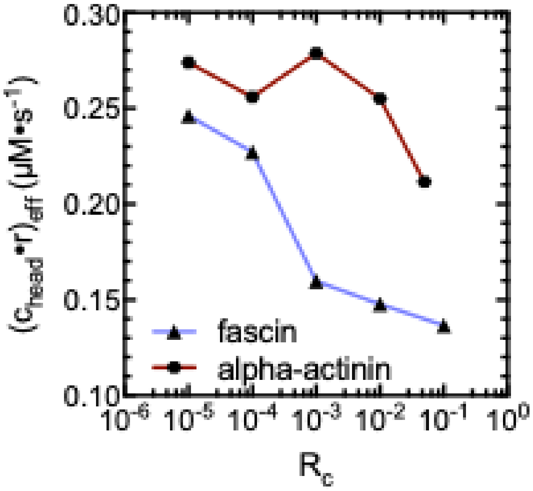

SFig 3. Effective unbinding rate plot. The effective unbinding rate for both crosslinker types under various R_{CL} conditions. All conditions have R_{myo} = 0.02. (Therefore c_{head} should approximately be the same)

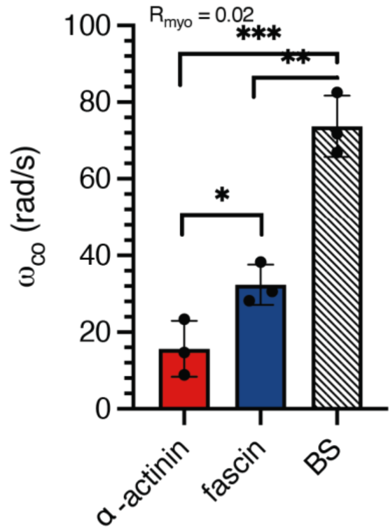

SFig 4. Crossover frequency. Crossover frequency ω_{co} for the three conditions in (c). N = 3 independent experiments for each condition. Welch's t-test is performed, $p_{f\text{-}a} = 0.0372$, $p_{BS\text{-}a} = 0.0008$, $p_{BS\text{-}f} = 0.003$.

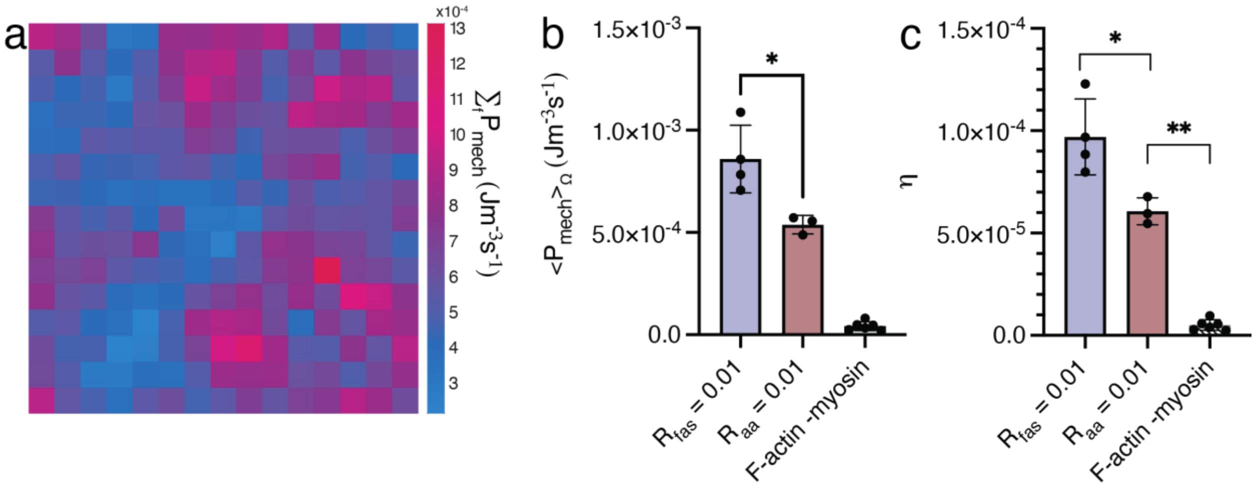

SFig 5. Mechanical dissipation and efficiency plot. a) Spatial map of the mechanical dissipation of the network summed over all frequencies for every window size. b) Averaged mechanical dissipation for R_{fas} and R_{aa} = 0.01, R_{myo} = 0.02, and F-actin without myosin case. c) Mechanical efficiency η for the three conditions. For b) and c), each point is an independent experimental run, the bars indicate the mean values and error bars are the s.t.d. of the mean. N = 4,3,6 respectively. For b), p_{fas-aa} = 0.0251, and for c), p_{fas-aa} = 0.0232, $p_{aa-actin}$ = 0.0027.

References

1. Kron, S. J., Toyoshima, Y. Y., Uyeda, T. Q. & Spudich, J. A. in *Methods in enzymology* Vol. 196 399-416 (Elsevier, 1991).
2. Falzone, T. T., Lenz, M., Kovar, D. R. & Gardel, M. L. Assembly kinetics determine the architecture of α-actinin crosslinked F-actin networks. *Nature communications* **3**, 861 (2012).
3. Pollard, T. D. Rate constants for the reactions of ATP-and ADP-actin with the ends of actin filaments. *The Journal of cell biology* **103**, 2747-2754 (1986).
4. Zheng, J. *et al*. A pico-calorimeter for cellular metabolism and antimicrobial susceptibility testing. *Proceedings of the National Academy of Sciences* **123**, e2603171123 (2026). https://doi.org:10.1073/pnas.2603171123

Supplementary movie captions

Supplementary Movie 1. 2D Crosslinked actin network contraction under myosin active stress. Fluorescent actin channel of fascin- (left) and α-actinin- (right) crosslinked network (R_{fas} and $R_{\alpha a} = 0.2$) contracting into astors and rupturing under myosin active stress. Scale bar is 50 µm.

Supplementary Movie 2. 3D fascin-crosslinked actomyosin network local deformation. Fascin-crosslinked (R_{fas} = 0.1) actin network exhibits local deformation overtime under myosin active stress. Scale bar is 10 µm.
